# Gut Dysbiosis and Intestinal Barrier Dysfunction Promotes IgA Nephropathy by Increasing the Production of Gd-IgA1

**DOI:** 10.3389/fmed.2022.944027

**Published:** 2022-07-07

**Authors:** Yuyan Tang, Yifan Zhu, Haidong He, Yinshun Peng, Ping Hu, Jiajun Wu, Weiqian Sun, Ping Liu, Yong Xiao, Xudong Xu, Minggang Wei

**Affiliations:** ^1^Department of Traditional Chinese Medicine, The First Affiliated Hospital of Soochow University, Suzhou, China; ^2^Department of Nephrology, Minhang Hospital, Fudan University, Shanghai, China; ^3^School of Public Health, Fudan University, Shanghai, China; ^4^Department of Emergency, Shanghai Ninth People's Hospital, Shanghai Jiaotong University School of Medicine, Shanghai, China

**Keywords:** IgA nephropathy, urine Gd-IgA1, intestinal mucosal barrier, intestinal microbiota, non-invasive biological marker

## Abstract

**Background:**

Immunoglobulin A nephropathy (IgAN) is the most common type of primary glomerular disease in adults worldwide. Several studies have reported that galactose-deficient IgA1 (Gd-IgA1) is involved in the pathogenesis of IgAN.

**Methods:**

Thirty-five patients with IgAN diagnosed with renal biopsy for the first time served as the experimental group, who were hospitalized in our department. Twenty normal healthy cases in the physical examination center of our hospital served as the control group. Then the levels of Gd-IgA1 in serum and urine, and intestinal mucosal barrier injury indexes [diamine oxidase (DAO), serum soluble intercellular adhesion molecule-1 (sICAM-1), D-lactate (D-LAC), and lipopolysaccharide (LPS)] and inflammatory factors [interleukin-6 (IL-6) and tumor necrosis factor-alpha (TNF-α)] in the serum samples were detected. Fecal samples were collected to detect intestinal microbiota using 16 s rDNA sequencing. Then, we assessed possible correlations among clinical and laboratory findings.

**Results:**

In patients with IgAN, the levels of Gd-IgA1 both in the serum and urine were higher than that of the healthy control. Furthermore, urine Gd-IgA1 level was positively correlated with the serum creatinine level, 24 h urine protein, and M, S, and T parameters in the Oxford classification. ROC curve analysis showed that urine Gd-IgA1 has a greater diagnostic value (AUC = 0.9714, 95% CI, 0.932–1; *P* < 0.0001) for IgAN. The best cutoff value for urine Gd-IgA1 was 0.745 ng·l/ml·μmol (sensitivity, 94%; specificity, 95%). The intestinal mucosal barrier damage indexes (DAO, sICAM-1, D-LAC, and LPS) were increased in the patients with IgAN, which were positively correlated with Gd-IgA1 levels (*P* < 0.05) both in serum and urine. The levels of inflammatory factors in the patients with IgAN were increased. 16 s rDNA analysis showed that the intestinal microbiota in these patients was disordered compared to that observed in the healthy subjects. *Actinobacteria, Bifidobacterium, Blautia, Bifidobacteriaceae, and Bifidobacteriales* were decreased and *Shigella* was increased in IgAN. The decreased populations of these flora were negatively and significantly correlated with urine Gd-IgA1 and the levels of DAO, sICAM-1, D-LAC, and LPS.

**Conclusion:**

The urine Gd-IgA1 levels may be a non-invasive biological marker for evaluating kidney injury in IgAN. Gut flora dysbiosis and intestinal barrier dysfunction may be involved in Gd-IgA1 expression.

## Introduction

Currently, IgAN is the most common type of primary glomerular disease in adults worldwide, accounting for 58.2% of the primary glomerular diseases in China (1, 2, 37). IgAN presents a variety of clinical features. Some patients progress rapidly without obvious symptoms, and about 30–40% of these cases develop the end-stage renal disease (ESRD) after 20–30 years, while some patients suffer no symptoms or just mild ones (36). IgAN is recognized as an immunological disease characterized by the deposition of IgA and Gd-IgA1 in the mesangial regions of the kidney. Elevated serum IgA and Gd-IgA1 levels have been reported in patients with IgAN compared with healthy controls (3). Gd-IgA1 has also been shown to further act as an autoantigen and induce the production of autoantibodies (4). It has been reported that the deposition of Gd-IgA1-containing immune complexes (IC) in the glomerular mesangial region with the assistance of IgA receptors leads to cell proliferation and excessive production of extracellular matrix, cytokines, and chemokines, resulting in glomerular injury (5).

However, the exact location of Gd-IgA1 production remains controversial. A recent genome-wide association study showed that most IgAN-associated loci are associated with immune-mediated inflammatory bowel disease, intestinal barrier maintenance, and response to intestinal pathogens (6). There is abundant evidence that intestinal mucosal immune response disorder is the main culprit in the development of IgAN (6, 7). Meanwhile, intestinal microbiome changes can increase antigen load and epithelial TLR recognition, thereby promoting B-cell classification conversion and IgA overproduction (8). This means that stable microbes can participate in intestinal dysbiosis, including the overgrowth of non-dominant or potentially harmful bacteria (9). However, the relationship between intestinal barrier function, microbiota, and Gd-IgA1 in IgAN is still unclear.

Although the current gold standard diagnostic and prognostic method for IgAN is a renal biopsy, it is not frequently performed in the real clinical field due to some limitations and concerns about complications. Thus, new biomarkers of IgAN are needed for non-invasive and more rapid diagnosis. There is emerging evidence that Gd-IgA1 plays a pivotal molecule in the pathogenesis of IgAN. However, few studies have investigated the role of Gd-IgA1 as a biomarker in IgAN. Therefore, this study aims to investigate the level of Gd-IgA1 in serum and urine of patients with IgAN and the validity of Gd-IgA1 as a biomarker for assessing renal injury in IgAN. Meanwhile, we also explored the probable mechanisms involved in the development of this disease.

## Materials and Methods

### Reagents

The renal puncture pathology was detected by the Pathology Department of Fudan University during the patient's hospitalization. The serum creatinine (Scr), blood urea nitrogen (BUN), and 24-h urinary protein (24-h-Upro) activities were measured using an ARCHITECT Automatic Biochemistry Analyzer. The serum Gd-IgA1 levels were detected by the KM55 ELISA kit, which was purchased from IBL, Japan. Batch Number (Lot No) 2E-117, Art. No. (Code No) 27600. DAO (CAT.no.sDH0135), D-LAC (CAT. No. sDH0134), sICAM-1 (CAT. No. sDH0131), LPS (CAT. No. sDH0133), TNF-α (CAT. No. SDH0001), and IL-6 (CAT. no. SDH0021) ELISA kits were purchased from Shanghai Siding Biotechnology Co., LTD. Quant-it Pico Green dsDNA Assay Kit was purchased from Invitrogen (P7589).

### Study Design

The study design included the prospective collection of samples from the inpatients visiting our hospital from January 2020 to December 2020. To avoid the effects of drugs and other hospital factors on the intestinal microorganisms, we included only newly diagnosed patients with IgAN who had not received any prior treatment and collected samples from inpatients before the initiation of any medication. The inclusion criteria were as follows: (1) Thirty-five patients with IgAN confirmed by renal biopsy pathology in our department for the first time were selected as the experimental group, (2) age ≥18 years, and (3) estimated glomerular filtration rate (eGFR) ≥60 ml/min/1.73 m^2^. Exclusion criteria were as follows: (1) use of glucocorticoids or immunosuppressants or receipt of a kidney transplant, (2) use of oral antibiotics, probiotics, prebiotics, or synbiotics within 2 months, or (3) history of tumor, blood system disease, or serious digestive disorders. Twenty healthy controls from a physical examination center were selected as the control group. Informed consent was obtained from the subjects, and the experimental protocol was approved by the Ethics Committee of the Minhang Hospital, Fudan University (Shanghai, China) (N0. 2018-010-01K).

### Serum, Urine, and Fecal Sample Collection

For all patients with glomerulonephritis, we collected 5 ml of fasting peripheral venous blood and morning urine samples, and the serum and urine were immediately separated. Samples were collected in Vacuette Serum Clot Tubes, centrifuged for 20 min at 3,000 rpm and supernatants aliquoted into small tubes and stored at −80 °C until use. Information regarding the characteristics of patients was recorded, and specimens for the subsequent tests were obtained from the samples diagnosed with IgAN and stored at −80 °C in a refrigerator. Five grams of fresh fecal sample was collected for intestinal micromicrobiota analysis.

### ELISA Analysis

Serum and urine samples were prepared as described above. According to the ELISA kit protocol, add 50 μL of sample and standard to each well, incubate at 37°C for 1 h, and then wash. Add 50 μL of enzyme-linked antibody to each well and incubate at 37°C for 0.5-1 h. Add 0.1 ml of temporary TMB substrate solution to each well, incubate at 37°C for 10–30 min, add 50 μL of stop solution to each well, and finally determine the absorbance (A) value at 450 nm with a microplate reader. The expression level was calculated by constructing a standard curve.

### Fecal Collection and DNA Extraction

The fresh feces of patients in the morning were collected in a sampling tube and stored at −80°C in a refrigerator. DNA was extracted from feces samples by the CTAB method, and DNA concentration and purity were detected using a NanoDrop ND-2000 ultra-fine spectrophotometer.

### PCR Amplification of Gene Sequence in V3–V4 Variable Region

Diluted genomic DNA was used as a template. The gene sequences of each bacterial 16 SrRNA gene reported in GenBank were compared by using the Chinese version of DNAMANV6 software, and the highly conservative V3–V4 region was selected. According to the basic principles of primer design, Primer 5.0 software was used to design a pair of primers. Shanghai Paisenuo Biological Technology Co., Ltd. was commissioned to synthesize 16 SrDNA sequence: F 5'- ACTCCTACGGGAGGCAGCA-3'; R 5'- GGACTACHVGGGTWTCTAAT-3'. The protocol for PCR analysis was as follows: pre-denaturation at 98°C for 2 min; denaturation at 98°C for 15 s; annealing at 55°C for 30 s; extension at 72°C for 30 s; 30 cycles; expansion at 72°C for 4 min; and storage at 4°C.

### Intestinal Microbiota Analysis

The bioinformatics analysis of the intestinal microbiome was performed using QIIME 2 2019.4 (10) with slight modifications according to the official tutorials (https://docs.qiime2.org/2019.4/tutorials/). Briefly, raw sequence data were demultiplexed using the demux plugin followed by primer cutting with the cutadapt plugin (11). Sequences were then quality filtered, denoised, and merged, and chimera was removed using the DADA2 plugin (12). Non-singleton amplicon sequence variants (ASVs) were aligned with MAFFT (13) and used to construct a phylogeny tree using fasttree2 (14). Alpha-diversity metrics and beta-diversity metrics (15) were estimated using the diversity plugin, and samples were rarefied to sequences per sample. Taxonomy was assigned to ASVs using the classify-sklearn native Bayes taxonomy classifier in a feature-classifier plugin (16) against the Greengenes 13_8 (99% OTU reference sequences) (17). The difference at the taxonomic level was calculated by linear discriminant analysis (LDA) effect size (LDA score >4, *p* < 0.05). Redundancy (RDA) analysis was performed to determine the relationship between intestinal microbial samples and intestinal mucosal damage indicators on the same two-dimensional sequence diagram. PICRUSt2 analysis was used to predict the potential functional units for intestinal microbiota.

### Statistical Analyses

GraphPad Prism 5.0 and SPSS22.0 software were used for mapping and statistical analysis. Non-normal distribution measurement data were expressed as median (minimum—maximum), and normal distribution measurement data were expressed as mean ± standard deviation (mean ± SD). Comparison between the two groups was performed by *t*-test. A non-parametric rank-sum test was used for the data with the abnormal distribution. Correlation analysis was calculated by the Spearman correlation coefficient. ROC curves were constructed to determine the prediction ability of IgAN. All the visualization work was performed by using the Genesys Cloud tool, a free online platform for data analysis (https://www.genescloud.cn).

## Results

### Baseline Characteristics of the Study Subjects

There were no statistically significant differences in gender ratio and age composition between the two groups (*P* > 0.05). As presented in Table 1, the levels of serum creatinine (Scr), blood urea nitrogen (BUN), 24-h urine protein, and cystatin C (CysC) in the IgA nephropathy group were significantly higher than those in the healthy control (HC) group, with a statistical significance (*P* < 0.05). The estimate glomerular filtration rate (eGFR) was significantly lower in the IgA nephropathy group than in the HC group. The frequency of the observation of pathological features (percentage) in 35 biopsies scored according to the Oxford Classification is summarized in Table 1.

**Table 1:** General data of experimental group and control group.

**Characteristics**	**IgAN**	**HCs**	***P*-value**
Gender (male/female)	18/17	11/9	0.799
Age (years)	40.6 ± 11.0	39.1 ± 8.4	0.596
BMI	24.1 ± 3.4	23.4 ± 3.3	0.438
course of disease (months)	6 (0.2–14)	–	–
Scr (μmol/L)	99.5 ± 26.8	67.3 ± 14.9	<0.0001
BUN (mmol/L)	5.4 ± 1.3	4.4 ± 0.5	0.001
eGFR	74.3 ± 22.6	103.1 ± 7.6	<0.0001
24 h Upro (g)	1.2 ± 1.1	0.12 ± 0.01	0.0001
Microscopic hematuria (/HP)	80.2 ± 155.5	–	–
M0/M1	17/18	–	–
E0/E1	12/23	–	–
S0/S1	9/26	–	–
T0/T1/T2	13/15/7	–	–
C0/C1/C2	11/24/0	–	–
ALB (g/L)	42.0 ± 3.1	43.8 ± 3.2	0.049
CysC (mg/L)	1.2 ± 0.3	0.6 ± 0.1	<0.0001
TG	1.6 ± 0.6	1.3 ± 0.5	0.074
TC	4.5 ± 1.1	4.5 ± 0.6	0.736
CRP	2.8 ± 3.1	2.3 ± 0.7	0.420

*Results are expressed as the mean ± SD values and ratio: SD standard deviation; BMI, body mass index; CysC, Cystatin C; BUN, blood urea nitrogen; Scr, creatinine; TG, triglyceride; TC, total cholesterol; eGFR, estimated glomerular filtration rate; Upro, urinary protein; CRP, C-reactive protein; ALB, albumin; MSETC: oxford classification of IgA nephropathy*.

### Gd-IgA1 Levels in Serum and Urine of the two Groups

**Figure 1:**
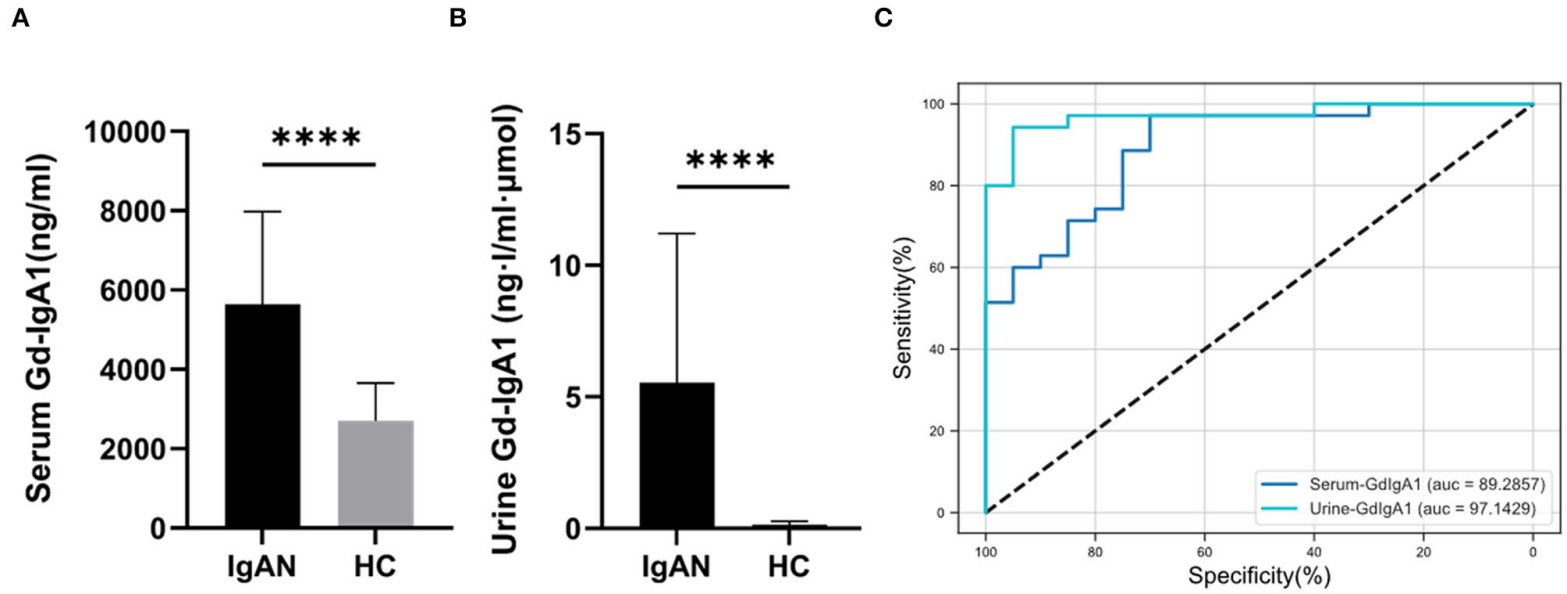
Serum and urine Gd-IgA1 levels. The level of serum **(A)** and urine **(B)** Gd-IgA1 levels were significantly higher in patients with IgAN compared to healthy and disease controls (****P < 0.0001). **(C)** ROC curve of the serum and urine levels of Gd-IgA1 in the exploration cohort with *P* < 0.05.

According to the literature (18), serum and urine Gd-IgA1 levels of the two groups were detected by the KM55 ELISA kit. Compared with the HC group (2,700 ± 952 ng/mL), the serum Gd-IgA1 levels of patients with IgAN (5,472 ± 2,334 ng/mL) were significantly increased (*t* = 5.068, *P* < 0.0001). The level of urinary Gd-IgA1 after correction in patients with IgAN (5.5 ± 5.6 ng·l/ml·μmol) was significantly higher than in the HC group (0.27 ± 0.37 ng·l/ml·μmol) (*t* = 4.100, *P* = 0.0001) (Figures 1A,B). ROC curve was used to assess the discrimination of the model, and the optimal cutoff point was obtained. According to the ROC curve (Figure 1C; Supplementary Table 1), urine Gd-IgA1 concentration could distinguish patients with IgAN from HC, with an area under the curve (AUC) of 0.9714 (95% CI, 0.932–1; *P* < 0.0001). The best cutoff value for urine Gd-IgA1 was 0.745 ng·l/ml·μmol (sensitivity, 94%; specificity, 95%). Serum Gd-IgA1 concentration could distinguish patients with IgAN from HC, with an AUC of 0.8929 (95% CI, 0.807–0.978; *P* < 0.0001). The best cutoff value for serum Gd-IgA1 level was 2,876.2 ng/mL (sensitivity, 97%; specificity, 70%). The ROC curve suggested that urine Gd-IgA1 has a greater diagnostic value for IgAN than serum Gd-IgA1.

### Gd-IgA1 Levels Were Associated With the Progression of IgA Nephropathy

To determine whether elevated Gd-IgA1 contributes to the progression of IgAN, we performed a correlation analysis between Gd-IgA1 and clinical indicators representing disease severity in patients with IgAN and summarized in Table 2. Correlation analysis showed that there was no correlation between serum Gd-IgA1 level and 24-h urine protein quantification (*P* > 0.05), but a positive correlation was observed with Scr (*r* = 0.453, *P* = 0.006). Notably, the urine Gd-IgA1 level was positively correlated with Scr (*r* = 0.574, *P* < 0.0001) and 24-h urine protein (*r* = 0.665, *P* < 0.0001). Furthermore, there was no correlation between microscopic hematuria and serum Gd-IgA1 level (*P* > 0.05), but a positive correlation was found with urine Gd-IgA1 (*r* = 0.442, *P* = 0.008). According to the 2017 Edition of Oxford Pathological Classification, namely the MEST-C score, the correlation analysis between Gd-IgA1 levels and M, E, S, T, and C parameters was also conducted. The results showed that there was no correlation between the serum Gd-IgA1 levels and M, S, E, and C parameters (*P* > 0.05), but a positive correlation was observed with T (*r* = 0.397, *P* = 0.018). The urine Gd-IgA1 levels were positively correlated with M (*r* = 0.551, *P* < 0.001), S (*r* = 0.368, *P* = 0.030), and T (*r* = 0.512, *P* = 0.002), suggesting that urine Gd-IgA1 was more correlated with the progression of IgAN. It is suggested that urine Gd-IgA1, as a non-invasive test index, provides a new idea for the early diagnosis and treatment of IgAN.

**Table 2:** Correlation between Gd-IgA1 and clinical indexes.

	**Serum-GdIgA1**		**Urine-GdIgA1**	
	***r***	***P*-value**	***r***	***P*-value**
Scr	0.453#	0.006^**^	0.574#	<0.0001^****^
24 h-Upro	0.188	0.278	0.665#	<0.0001^****^
Microscopic hematuria (/HP)	0.375	0.027	0.442#	0.008^**^
M	0.301	0.079	0.551#	0.001^**^
S	−0.178	0.307	0.368	0.03^**^
E	−0.03	0.864	−0.15	0.389
T	0.397	0.018^**^	0.512#	0.002^**^
C	0.062	0.722	0.017	0.923

*Scr, serum creatinine; 24-h-Upro, 24-h urine protein; MSETC: Oxford Classification of IgA nephropathy; #r > |0.4|*;

* *P < 0.05*;

** *P < 0.01*;

**** *P < 0.0001*.

### Serum Inflammatory Cytokine Levels in Both Groups

**Figure 2:**
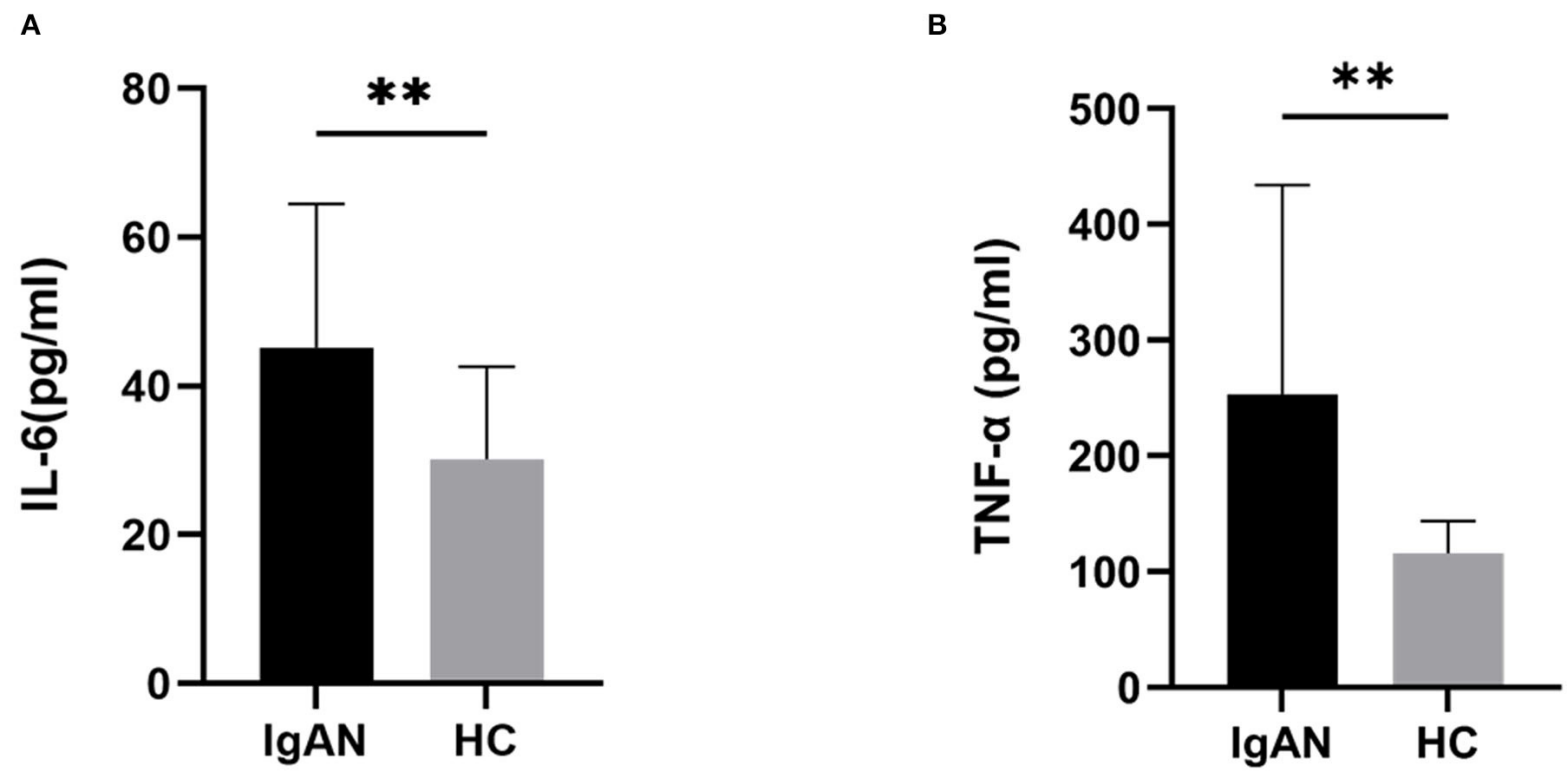
Serum inflammatory cytokine levels in both groups. The serum levels of IL-6, and TNF-α from patients with IgAN were assayed. Higher levels of serum IL-6 **(A)**, and TNF-α **(B)** were detected in patients with IgAN compared with healthy subjects. Data are from at least three independent experiments. Data represent the mean ± sd values. *P < 0.05, **P < 0.01.

We used an ELISA kit to detect the levels of inflammatory factors (IL-6 and TNF-α) in the serum samples of the two groups. The results showed that the level of TNF-α in the patients with IgAN (253.2 ± 181.0 pg/mL) was significantly higher than that in the control group (115.7 ± 27.7 pg/mL) (*t* = 3.360, *P* = 0.0015). The level of IL-6 (45.2 ± 19.3 pg/mL) in the patients with IgAN was significantly higher than that in the control group (30.2 ± 12.5 pg/mL) (*t* = 3.116, *P* = 0.0030) (Figure 2). It is suggested that patients with IgAN patients were in a systemic microinflammation state.

### Different Bacterial Diversity and Composition Between Two Groups

**Figure 3:**
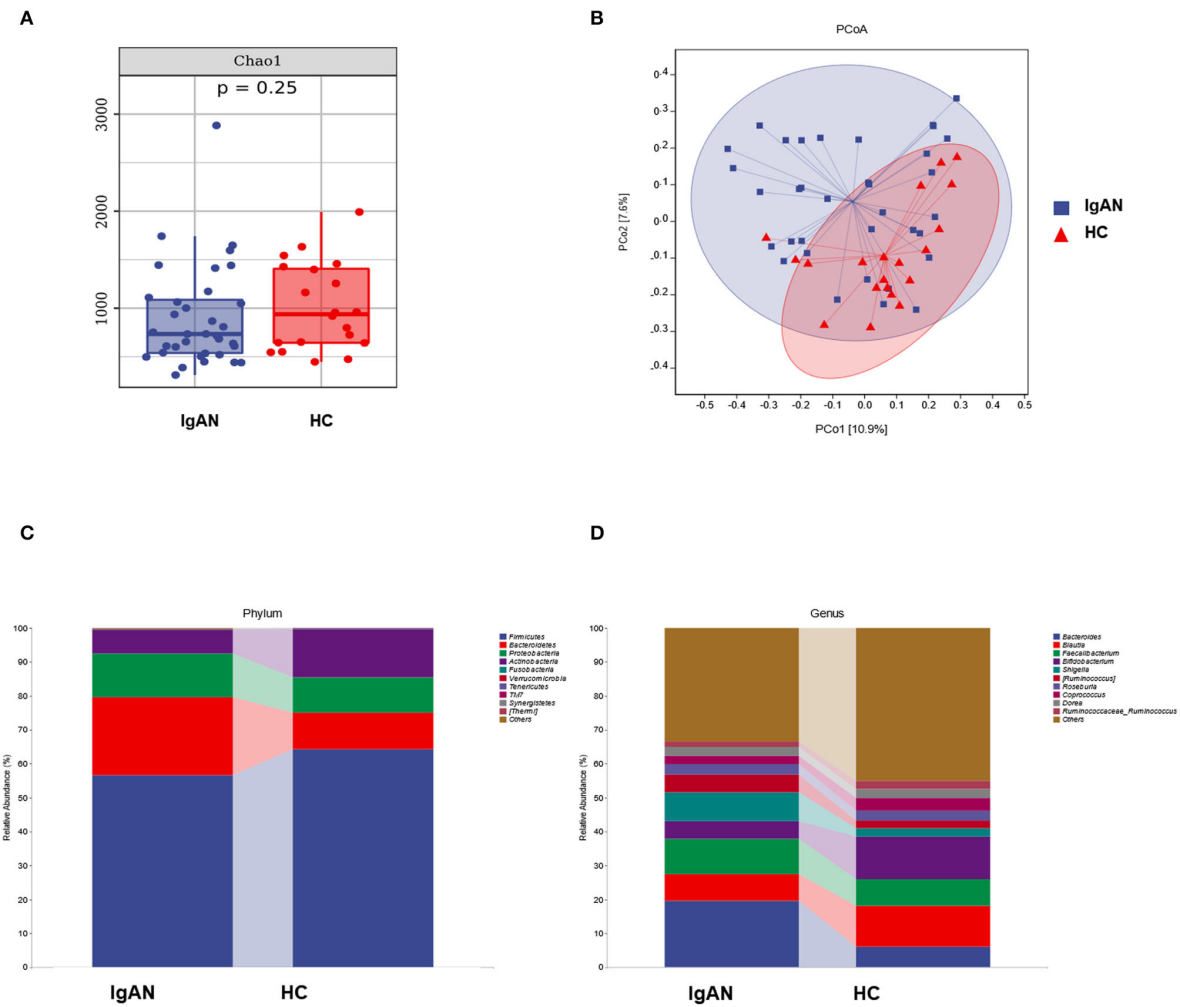
The analysis of intestinal microbiota of the two groups. **(A)** The alpha diversity, **(B)** PCoA analysis, and **(C,D)** the microbiota structure of the two groups at the level of phylum and genus.

o explore the mechanism of the increased production of Gd-IgA1 in the patients with IgAN, we further analyzed the bacterial diversity and communities of the two groups by 16S rDNA sequence analysis. Even though there was no significance, Chao 1, an index representing the α-diversity, had an increasing tendency in the HC group than in the IgAN group (Figure 3A). Next, we applied PCoA analysis to represent β-diversity (Figure 3B). Through PCoA analysis, we found that the intestinal flora of the two groups can be clearly separated as a whole and exhibited significant differences.

There were also differences in the bacterial community structure between the two groups. At the phylum level, *Bacterioidetes* (IgAN: 22.99%, HC: 10.72%, *P* = 0.0363), *Proteobacteria* (IgAN: 12.87%, HC: 10.36%, *P* = 0.6694), *Fusobacteria* (IgAN: 0.14%, HC: 0.09, *P* = 0.6011), *Firmicute* (IgAN: 56.60%, HC: 64.33%, *P* = 0.2345), and *Actinobacteria* (IgAN: 7.09%, HC: 14.32% *P* = 0.0704) were the top five phyla. At the genus level, *Bacteroides* (IgAN: 19.59%, HC: 6.13%, *P* = 0.0173), *Faecalibacterium* (IgAN: 10.40%, HC: 7.80%, *P* = 0.4610), *Shigella* (IgAN: 8.40%, HC: 2.48%, *P* = 0.1555), *Blautia* (IgAN: 7.93%, HC: 11.97%, *P* = 0.0387), and *Bifidobacterium* (IgAN: 5.25%, HC: 12.65%, *P* = 0.0483) were the top five genera (Figures 3C,D).

**Figure 4:**
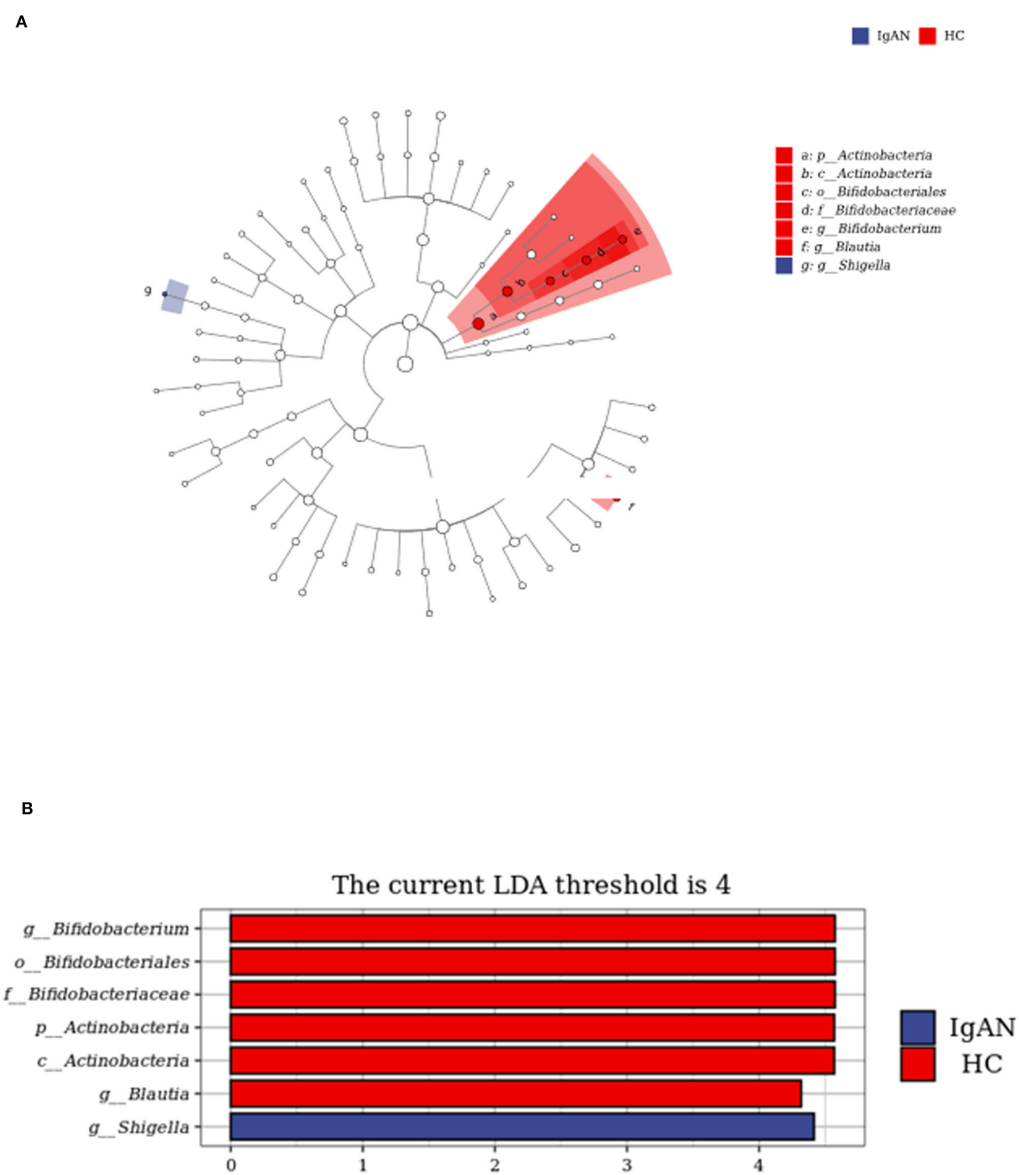
LEfSe analysis to identify biomarkers of IgAN. **(A)** Cladogram of LEfSe analysis. **(B)** Histogram of LEfSe analysis.

To identify the biomarkers of IgAN, we performed a taxonomic assignment of the sequences and analyzed the taxonomic profile of each sample using the LEfSe algorithm, which showed significant differences between groups at all the taxonomic levels (Figure 4). We applied a strict filter condition on the LEfSe algorithm, that is, LDA >4. The cladogram showed the hierarchy of different biomarkers and the evolutionary relationship between them (Figure 4A). The histogram showed that *p_Actinobacteria, c_Actinobacteria, g*_ *Bifidobacterium, g*_*Blautia, f* _*Bifidobacteriaceae*, and *o*_*Bifidobacteriales* were decreased in the IgAN group (Figure 4B). Notably, all these biomarkers belonged to the phylum *Actinobacteria*, while only *g* _*Shigella* was found to be increased in IgAN.

### Functional Analysis of Gut Microbiota Predicted by PICRUSt2

**Figure 5:**
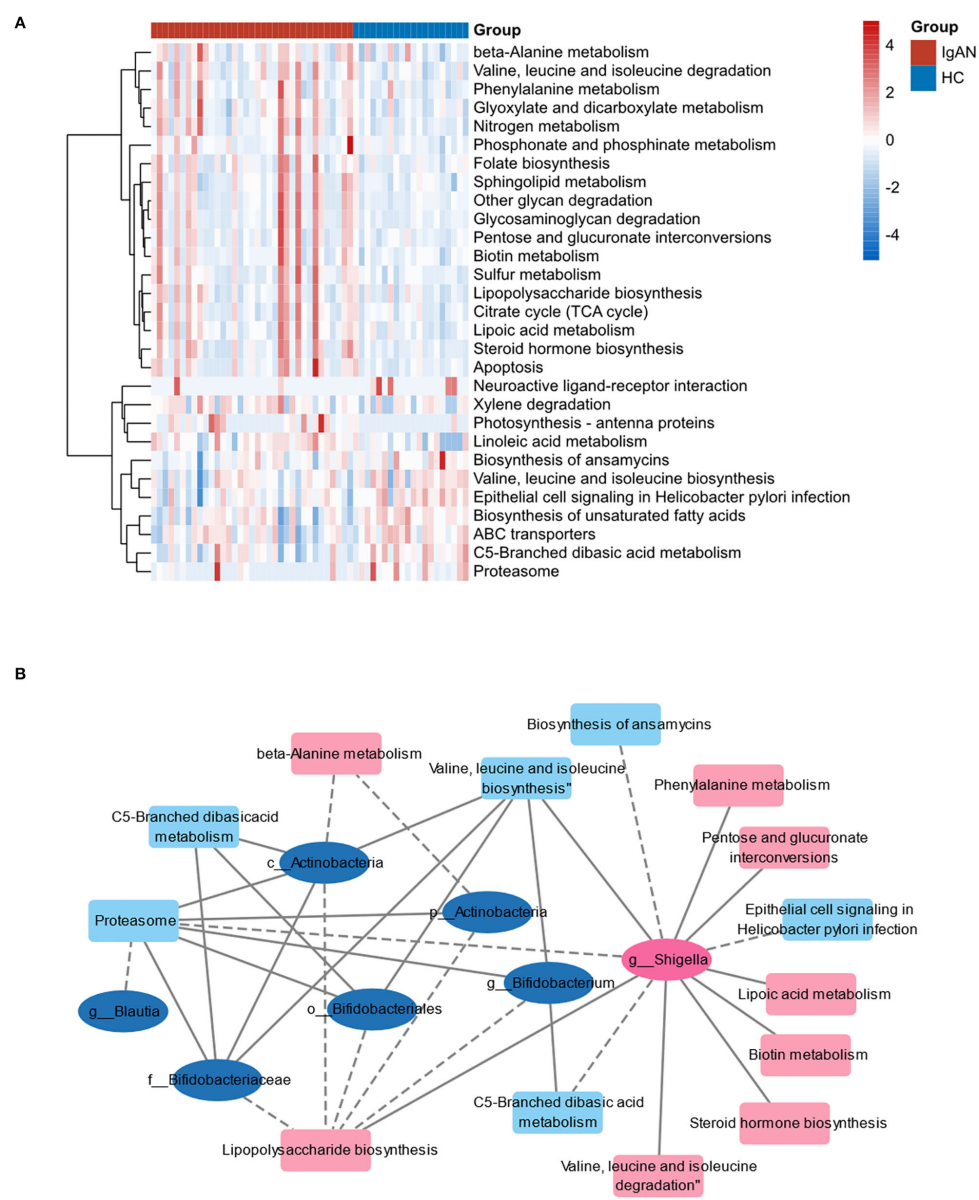
Functional analysis of gut microbiota predicted by PICRUSt2. **(A)** Heat map of the predictive metabolic pathways of two groups. **(B)** The correlation network of predictive metabolic pathways and changed microbiome. The ellipses represent the bacteria. The squares represent the metabolic pathways. Pink marks indicate that the bacteria or pathway is upregulated in IgAN and blue marks indicate that they are downregulated. The line between two nodes indicates correlation: the solid line indicates positive correlation, and the dotted line indicates negative correlation.

Referring to the known microbial genome data, PICRUSt2 analysis was used to predict the potential composition of flora genes or functional units for intestinal microbiota based on 16S rRNA sequencing results. The analysis of Level 3 KEGG function classes revealed that 14 metabolic pathways differed statistically between the IgAN and HC groups (*P* < 0.05). These altered metabolic pathways are shown in the heatmap (Figure 5A). The correlation coefficients between the changed microbiome and predictive metabolic pathways were then calculated and merged into a network (Figure 5B). Biotin metabolism; pentose and glucuronate interconversions; lipoic acid metabolism; lipopolysaccharide biosynthesis; valine, leucine, and isoleucine degradation; phenylalanine metabolism; steroid hormone biosynthesis; and valine, leucine, and isoleucine biosynthesis were positively related to *g__Shigella*, which were increased in the IgAN group. However, the biosynthesis of ansamycins, metabolism of C5-branched dibasic acid, metabolism and sporulation of proteasome ether lipids, and epithelial cell signaling in Helicobacter pylori infection were negatively related to *g__Shigella* (*P* < 0.05, *r* > |0.4|).

### Intestinal Barrier Function Analysis

**Figure 6:**
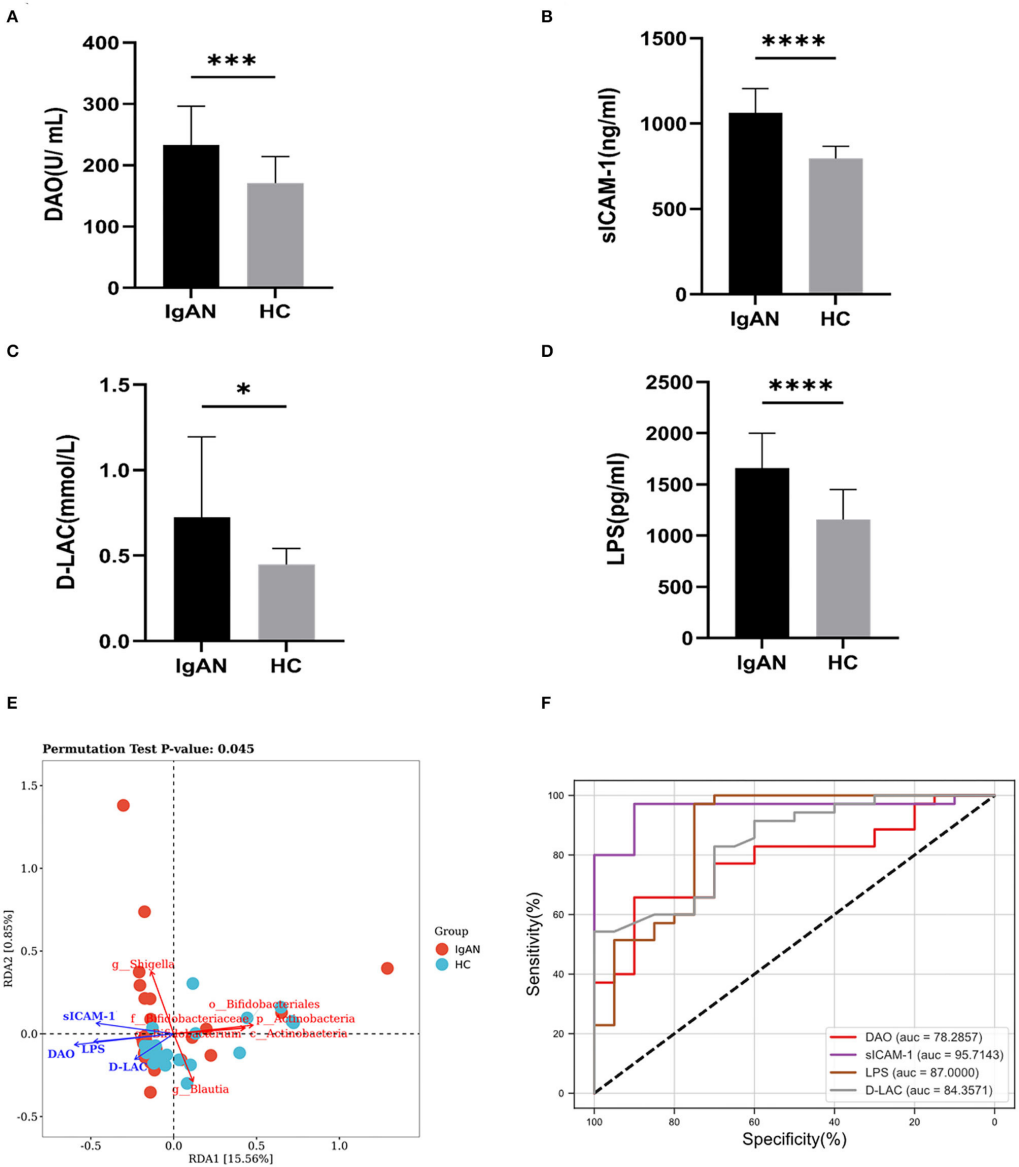
Intestinal mucosal barrier injury indexes. The levels of intestinal barrier injury indicators: DAO, sICAM-1, D-LAC, and LPS in peripheral blood were assayed. The levels of DAO **(A)**, sICAM-1 **(B)**, D-LAC **(C)**, and LPS **(D)** were significantly higher in patients with IgAN compared to healthy and disease controls (*P < 0.05, ***P < 0.001, ****P < 0.0001). **(E)** Redundancy analysis (RDA) was constructed to detect the correlation between intestinal barrier function and gut microbiota. **(F)** ROC curve of the levels of intestinal barrier injury indicators in the exploration cohort with *P* < 0.05.

To explore the microinflammatory status and the mechanism of Gd-IgA1 production in a patient with IgAN, we detected the levels of intestinal barrier injury indicators, that is, DAO, D-LAC, sICAM-1, and LPS in the peripheral blood. Compared with the HC group (170.7 ± 43.4 U/mL), the DAO level in the IgAN group (233.2 ± 63.1 U/ml) was higher (*t* = 3.925, *P* = 0.0003) (Figure 6A). The expression level of sICAM-1 was higher in the IgAN patients (1063.3 ± 141.9 ng/mL) than that of the healthy group (797.5 ± 69.7 ng/ml) (*t* = 7.832, *P* < 0.0001) (Figure 6B). The D-LAC level of IgAN patients (0.72 ± 0.47 mmol/L) was higher than that of the healthy group (0.45 ± 0.09 mmol/L) (*t* = 2.566, *P* = 0.0131) (Figure 6C). LPS levels were higher in IgAN patients (1,658.8 ± 342.9 pg/ml) than in the healthy group (1,160.2 ± 291.6 pg/ml) (*t* = 5.465, *P* < 0.0001) (Figure 6D). The results indicated that the barrier function of intestinal mucosa was impaired in the IgAN patients.

Redundancy analysis (RDA) was performed to detect the correlation between intestinal barrier function and gut microbiota. The significance of each metal and RDA axis in the RDA model was checked by a permutation test (*P* = 0.045). Table 3 showed that DAO, sICAM-1, D-LAC, and LPS were significant variables (*P* < 0.05). The direction and length of the element vectors and the coordinates of the seven biomarkers in the biplot revealed the relationship between them. As shown in Figure 6, DAO had the most significant effect on the composition of the bacterial community, as shown by the longest vector length. In addition, *p_Actinobacteria, c_Actinobacteria, Bifidobacterium, Blautia, Bifidobacteriaceae*, and *Bifidobacteriales* showed a relatively negative correlation with the levels of DAO, sICAM-1, D-LAC, and LPS (*P* < 0.05). *Shigella* was positively correlated with the level of DAO, sICAM-1, D-LAC, and LPS (*P* < 0.05).

**Table 3:** Importance of the variables in the RDA models for the microbial community.

	**RDA1**	**RDA2**	**r2**	**Pr (>r)**
LPS	−0.999990452	−0.004369947	0.117611681	0.041^*^
sICAM-1	−0.990050651	0.140711435	0.11851879	0.036^*^
D-LAC	−0.931901166	−0.362712307	0.039985826	0.21
DAO	−0.999917742	−0.012826081	0.181653237	0.003^**^

* *P < 0.05*;

** *P < 0.01*.

According to the ROC curve (Figure 6F; Supplementary Table 2), sICAM-1 could best distinguish IgAN patients from HC, with an AUC of 0.9571 (95% CI, 0.901–1; *P* < 0.0001). The best cutoff value was found to be 883.3 ng·l/ml (sensitivity, 94%; specificity, 95%).

### Relationship Between Gd-IgA1, Mucosal Barrier Injury Indexes, and Gut Microbiota

**Figure 7:**
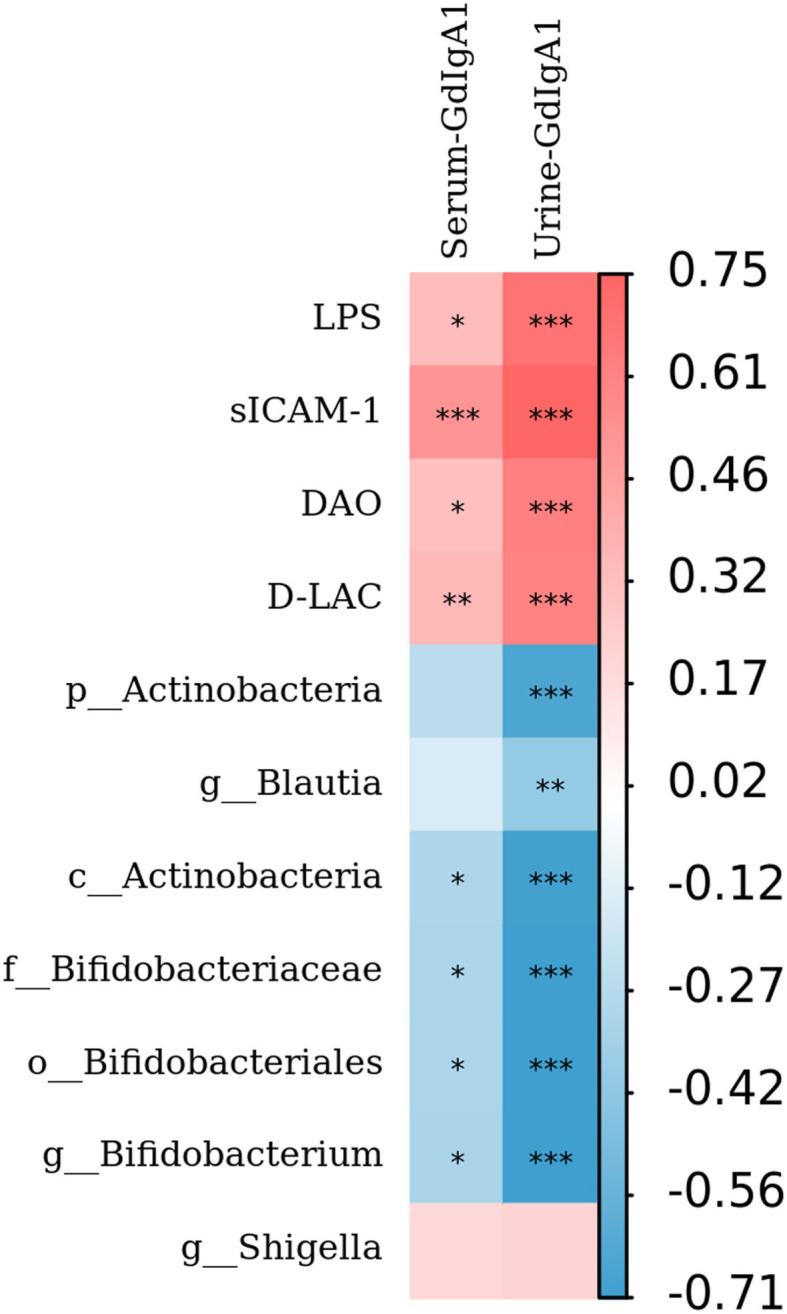
Relationship between Gd-IgA1, intestinal mucosal barrier injury indexes, and bacterial biomarkers. The level of Gd-IgA1, the levels of intestinal mucosal barrier injury indexes (DAO, sICAM-1, D-LAC, and LPS), and bacterial biomarkers in patients with IgAN were analyzed by Spearman correlation analysis. The red square represents the correlation coefficient (*r*) >0, while the blue square represents the *r* < 0. *P < 0.05, **P < 0.01, ***P < 0.001.

To further analyze the mechanism of Gd-IgA1 production, the level of Gd-IgA1, the levels of intestinal mucosal barrier injury indexes (DAO, sICAM-1, D-LAC, and LPS), and bacterial biomarkers in IgAN patients were analyzed by Spearman correlation analysis. The results showed that both serum and urine Gd-IgA1 levels were positively correlated with the intestinal barrier injury indexes in IgAN patients, particularly sICAM-1 (*P* < 0.001), suggesting that the expression level of Gd-IgA1 was correlated with the impairment of intestinal barrier function (Figure 7). In addition, urine Gd-IgA1 level was negatively correlated with *Actinobacteria, Bifidobacterium, Bifidobacteriaceae*, and *Bifidobacteriales* (*P* < 0.05).

## Discussion

Immunoglobulin A nephropathy is the most common primary glomerular disease worldwide, which leads to end-stage kidney disease (ESKD) in 20–40% of the cases (19). Recent studies have shown that Gd-IgA1 is called “nephrogenic IgA” and is the key pathogenic factor of IgAN. So far, the mechanism underlying the pathogenesis remains unclear (20). In this study, serum and urine levels of Gd-IgA1 were both detected, and the clinical relevance of Gd-IgA1 in patients with IgAN was investigated. The results showed that serum and urine Gd-IgA1 levels in patients with IgAN were higher than those in the control group, and the ROC curve showed that urine Gd-IgA1 was more valuable for the diagnosis of IgAN than serum Gd-IgA1. Urine Gd-IgA1 concentration could distinguish IgAN patients from HC, with an area under the curve (AUC) of 0.9714. The best cutoff value for urine Gd-IgA1 was found to be 0.745 ng·l/ml·μmol (sensitivity, 94%; specificity, 95%). Correlation analysis showed that there was no correlation between serum Gd-IgA1 level and 24-h urine protein levels in patients with IgAN, but a positive correlation was observed with serum creatinine. There was no correlation between serum Gd-IgA1 level and M, E, S, and C parameters, but a positive correlation was noticed with T. The urine Gd-IgA1 levels were positively correlated with 24-h urine protein, microscopic hematuria, and creatinine level, as well as with M, S, and T parameters, suggesting a better clinical relevance between urine Gd-IgA1 and IgAN. These results suggested that the urine Gd-IgA1 levels have a greater value for evaluating kidney injury in patients with IgAN. Previous studies have also suggested that increased serum Gd-IgA1 levels in patients with IgAN were related to the prognosis of patients (21). But these studies did not investigate the correlation between urinary Gd-IgA1 levels and IgAN. Therefore, this study found that the level of Gd-IgA1 in the urine of patients with IgAN was increased, which had a greater diagnostic value for IgAN than serum Gd-IgA1.

Although significant progress has been made since Berger et al. first described IgAN in 1968, the pathogenesis of the disease is not fully understood (35). Previous studies reported that the incidence of IgAN and its severity are closely associated with intestinal dysbacteriosis. Imbalance in intestinal microbiota may lead to changes in the intestinal barrier, which is conducive to the absorption of toxins into the body by the intestinal mucosa and activation of intestinal mucosal lymphoid tissue (GALT), thus inducing increased levels of Gd-IgA1 and eventually leading to the deposition of IgA1 on the glomerular mesangial area. This is called the “gut–kidney axis” of IgAN (6, 22). Intestinal microbes and their derived metabolites have been considered to have a significant impact on immune homeostasis (23, 24). These gut microbes maintain the integrity of the epithelial barrier and shape the intestinal immune system, thus balancing host defense through microbial metabolites, composition, and attachment to host cells (25). At the same time, intestinal mucosa contains different types of immune cells, which are involved in maintaining healthy intestinal microbes and enhancing epithelial barrier function (26). Won et al. (27) found that intestinal microbiota can activate GALT to secrete type I interferon, upregulate the expression of B-cell activating factors (BAFF and APRIL), and activate the IgA immunoglobulin category transformation and recombination of B cells, and these antigen-sensitized B cells migrate to the intestinal lamina. Further, these B cells migrate to the lamina propria and synthesize dimer IgA1. It has also been observed that the LPS of Gram-negative bacteria could activate TLR4 in the cultured IgAN peripheral blood B lymphocytes. More specifically, LPS strongly inhibited the mRNA expression of core 1β 3-galactosyl transferase-specific molecular chaperone (Cosmc), leading to the overproduction of polymerized Gd-IgA1 (28).

However, the precise correlation between Gd-IgA1 and gut microbiota in patients with IgAN is uncertain. The results of this study showed that compared with the control group, the levels of TNF-α and IL-6 in IgAN patients were significantly increased, and the bacterial community analysis showed that the diversity in IgAN patients decreased. There were significant differences between the two groups in terms of the bacterial community structure. At the level of phylum, the proportion of *Bacteroidetes, Proteobacteria*, and *Fusobacteria* increased in IgAN, while the proportion of *Firmicute* and *Actinobacteria* declined. At the genus level, the proportion of *Bacteroidete*s, *Faecalibacterium*, and *Shigella* increased in IgAN patients, while the proportion of *Blautia* and *Bifidobacterium* declined. To identify the biomarkers of the disease, we performed a taxonomic assignment of the sequences and analyzed the taxonomic profile of each sample using the LEfSe algorithm, which showed significant differences between groups at all taxonomic levels. We applied a strict filter condition on the LEfSe algorithm, that is, LDA >4. The cladogram showed the hierarchy of different biomarkers and the evolutionary relationship between them. The histogram showed that *p_Actinobacteria, c_Actinobacteria, g_ Bifidobacterium, g_Blautia, f_Bifidobacteriaceae*, and *o_Bifidobacteriales* were decreased in IgAN. Notably, all these biomarkers belonged to the phylum *Actinobacteria*. Only *g _Shigella* was found to be increased in IgAN. The above results indicated that the intestinal microbiota of IgAN patients was dysregulated.

Normal intestinal microbiota plays an important role in maintaining intestinal mucosal barrier function. The pathogenesis and progression of IgAN are closely related to the intestinal mucosal barrier. The role of intestinal mucosal barrier function in the pathogenesis and development of chronic kidney disease has been paid more and more attention. The intestinal mucosal barrier plays an important role in protecting the body from food antigens, pathogenic microorganisms, and their harmful metabolites (29). It was found that the intestinal mucosal barrier was destroyed in the IgAN rat model, and the drug use to protect the intestinal mucosa could reduce the pathological damage associated with IgAN to rat kidney tissue: IgA deposition in mesangial area, 24-h urine protein, and microscopic hematuria were found to be decreased (30). At the same time, changes in the intestinal microbiome can increase antigen load and epithelial TLR recognition, thereby promoting B-cell classification conversion and IgA overproduction (8). Therefore, impaired intestinal mucosal barrier function may be involved in Gd-IgA1 production.

We can evaluate the intestinal mucosal barrier function of patients with IgAN to study the mechanism of Gd-IgA1 production. However, it is still difficult to directly observe the intestinal barrier function. At present, the barrier function of the intestinal mucosa is indirectly reflected by the detection of DAO, sICAM-1, D-LAC, and LPS in the peripheral blood (31). DAO is a highly active intracellular enzyme found in the cytoplasm of villous cells in the upper layer of intestinal mucosa. When the intestinal mucosal epithelial cells are injured, more amount of DAO will be released into the intracellular space and further enter the intestinal intercellular space and lymphatic vessels, which can increase DAO in plasma and keep DAO activity stable in peripheral blood (32). D-LAC is the final metabolic product of gastrointestinal bacteria, which can also be produced by various intestinal bacteria. Mammals do not have the enzyme system to rapidly metabolize and degrade it. When intestinal mucosa is damaged and its permeability is increased, D-LAC secretion is increased and enters the peripheral blood circulation. Therefore, the detection of DAO and D-LAC can reflect intestinal injury and repair. sICAM-1 belongs to the immunoglobulin superfamily, and its role in the pathogenesis of inflammatory bowel disease (IBD) has attracted attention in recent years. sICAM-1 is usually expressed at low levels in vascular endothelial cells, lamina propria of the intestinal mucosa, and mononuclear macrophages in lymph nodes in normal intestinal tissue. In IBD intestinal tissue, the expression and distribution of sICAM-1 are significantly increased and are closely related to the degree of tissue inflammation (33, 34). Therefore, detection of serum sICAM-1 level may become an important indicator for monitoring the functional status of intestinal mucosal (33).

In this study, DAO, sICAM-1, D-LAC, and LPS were quantitatively measured in 35 IgAN patients and 20 healthy subjects. The results showed that the levels of DAO, sICAM-1, D-LAC, and LPS in the peripheral blood of patients with IgAN were also higher than those in the control group, suggesting that the intestinal mucosal barrier function was impaired in patients with IgAN. Redundancy analysis (RDA) was constructed to detect the correlation between intestinal barrier function and gut microbiota. Results have shown that DAO had the most significant effect on the composition of the bacterial community, as shown by the longest vector length. In addition, *p_Actinobacteria, c_Actinobacteria, Bifidobacterium, Blautia, Bifidobacteriaceae*, and *Bifidobacteriales* have a relatively negative correlation with the levels of DAO, sICAM-1, D-LAC, and LPS and urine Gd-IgA1 levels. *Shigella* was positively correlated with the levels of DAO, sICAM-1, D-LAC, and LPS. The above results suggested that alterations in the bacterial community in patients with IgAN might be related to the impairment of intestinal barrier function. Therefore, we further analyzed the correlation between intestinal mucosal barrier injury index and Gd-IgA1 levels, and the results showed that the levels of DAO, sICAM-1, D-LAC, and LPS were positively correlated with serum and urine Gd-IgA1 levels. It is suggested that intestinal microbiota and intestinal mucosal barrier function are impaired, leading to an inflammatory reaction, which subsequently induces increased production of Gd-IgA1.

In conclusion, this study confirmed that the level of Gd-IgA1 was positively correlated with the severity of IgAN in patients, especially the urinary Gd-IgA1 level. The urine Gd-IgA1 can be used as a non-invasive biological indicator for IgAN. Gut dysbiosis and impaired intestinal mucosal barrier function may be involved in Gd-IgA1 expression, which will provide new ideas for the diagnosis and treatment of IgAN.

## Data Availability Statement

The raw data have been uploaded in the Supplementary Material. The raw data of 16S sequencing for this study can be found in the (PRJNA828554) in NCBI.

## Ethics Statement

All samples have obtained informed consent of the subjects and have been approved by the Ethics Committee of the Minhang Hospital, Fudan University (Shanghai, China) (No. 2018-010-01K). Written informed consent to participate in this study was provided by the participants' legal guardian/next of kin.

## Author Contributions

YT, MW, YZ, and HH conceived the study and designed the study protocol. YT, YZ, HH, YP, and XX performed the experiments and wrote the manuscript. PL, PH, WJ, and WS analyzed the data. YT prepared the manuscript, with editing and revision by all authors. All authors contributed to the article and approved the submitted version.

## Funding

The study was supported by the Suzhou Science and Technology Bureau of the application of the basic research project (No. SYS2020119), Jiangsu Province Traditional Chinese Medicine Science and Technology Development Plan Project (No. MS2021098), the Ministry of Education Industry-University Cooperation Collaborative Education Project (No. 202102242003), and Shanghai Minhang District High-Level specialty backbone physician training program funding project (No. 2020MZYS19).

## Conflict of Interest

The authors declare that the research was conducted in the absence of any commercial or financial relationships that could be construed as a potential conflict of interest.

## Publisher's Note

All claims expressed in this article are solely those of the authors and do not necessarily represent those of their affiliated organizations, or those of the publisher, the editors and the reviewers. Any product that may be evaluated in this article, or claim that may be made by its manufacturer, is not guaranteed or endorsed by the publisher.
