# Supplementary material for: Gut Dysbiosis and Intestinal Barrier Dysfunction Promotes IgA Nephropathy by Increasing the Production of Gd-IgA1
Source: Front Med (Lausanne). 2022 Jul 7;9:944027. doi: 10.3389/fmed.2022.944027 (PMC9302483; doi:10.3389/fmed.2022.944027)
Supplement: Supplementary file 1 [file Table_1.DOCX]

Supplementary Material

# Supplementary Tables

## Supplementary Table 1

**Table S1. Evaluation of the Gd-IgA1 levels combinations (in accordance with Figure 1C)**

| **Index** | **Serum Gd-IgA1** | **Urine Gd-IgA1** |
| --- | --- | --- |
| **AUC** | 89.28571429 | 97.14285714 |
| **P-value** | <0.0001 | <0.0001 |
| **Best Cut-off Value** | 2876.1953 | 0.744800423 |
| **Sensitivity** | 97.14285714 | 94.28571429 |
| **Specificity** | 70 | 95 |
| **Accuracy** | 87.27272727 | 94.54545455 |
| **Precision** | 85 | 97.05882353 |
| **Youden Index** | 167.1428571 | 189.2857143 |
| **Confidence interval** | 0.807 | 0.932 |
|  | 0.978 | 1 |

## Supplementary Table 2

**Table S2. Evaluation of the levels of intestinal mucosal barrier injury indexes combinations (in accordance with Figure 6F)**

| **Index** | **DAO** | **sICAM-1** | **LPS** | **D-LAC** |
| --- | --- | --- | --- | --- |
| **AUC** | 78.28571429 | 95.71428571 | 87 | 84.35714286 |
| **P-value** | 0.0005 | <0.0001 | <0.0001 | <0.0001 |
| **Best Cut-off Value** | 219.6 | 883.3 | 1255.375 | 0.6225 |
| **Sensitivity** | 65.71428571 | 97.14285714 | 97.14285714 | 54.28571429 |
| **Specificity** | 90 | 90 | 75 | 100 |
| **Accuracy** | 74.54545455 | 94.54545455 | 89.09090909 | 70.90909091 |
| **Precision** | 92 | 94.44444444 | 87.17948718 | 100 |
| **Youden Index** | 155.7142857 | 187.1428571 | 172.1428571 | 154.2857143 |
| **Confidence interval** | 0.662 | 0.901 | 0.761 | 0.741 |
|  | 0.904 | 1 | 0.979 | 0.946 |
